# KAP1 is a Novel Substrate for the Arginine Methyltransferase PRMT5

**DOI:** 10.3390/biology4010041

**Published:** 2015-01-09

**Authors:** Roberta di Caprio, Michela Ciano, Giorgia Montano, Paola Costanzo, Elena Cesaro

**Affiliations:** Department of Molecular Medicine and Medical Biotechnology, University of Naples Federico II, via S. Pansini 5, Naples 80131, Italy; E-Mails: rob.dicaprio@libero.it (R.C.); michelaciano@libero.it (M.C.); giorgia.montano@unina.it (G.M.); elenacesaro@virgilio.it (E.C.)

**Keywords:** KRAB-associated protein 1 (KAP1), protein-protein interaction, post-translational modifications, protein arginine methyltransferase

## Abstract

KRAB-associated protein 1 (KAP1), the transcriptional corepressor of Kruppel-associated box zinc finger proteins (KRAB-ZFPs), is subjected to multiple post-translational modifications that are involved in fine-tuning of the multiple biological functions of KAP1. In previous papers, we analyzed the KAP1-dependent molecular mechanism of transcriptional repression mediated by ZNF224, a member of the KRAB-ZFP family, and identified the protein arginine methyltransferase PRMT5 as a component of the ZNF224 repression complex. We demonstrated that PRMT5-mediated histone arginine methylation is required to elicit ZNF224 transcriptional repression. In this study, we show that KAP1 interacts with PRMT5 and is a novel substrate for PRMT5 methylation. Also, we present evidence that the methylation of KAP1 arginine residues regulate the KAP1-ZNF224 interaction, thus suggesting that this KAP1 post-translational modification could actively contribute to the regulation of ZNF224-mediated repression.

## 1. Introduction

KAP1 is a well-known transcriptional corepressor of KRAB-ZFPs. The N-terminus of KAP1 contains a tripartite motif composed of a Ring finger, two B-box zinc fingers and a coiled-coil (RBCC) domain; this motif binds the KRAB repression module as a homotrimer. It has been shown that transcriptional repression mediated by KRAB-ZNFs depends on KAP1 as well as on its interacting partners [1,2]. Indeed, the C-terminus tandem PHD and the bromodomain of KAP1 act as scaffold domains recruiting histone deacetylases, histone methylases and other chromatin modifiers to promoters of target genes. Furthermore, the central region of KAP1 includes the binding domain of heterochromatin protein 1 (HP1), involved in heterochromatin packaging. In this way, KAP1 coordinates the assembly of components required for gene silencing by forming a facultative heterochromatin environment [3,4]. Moreover, KAP1 is subjected to several post-translational modifications, including SUMOylation and phosphorylation [5,6]. These modifications, required for the repressive activity and for the recruitment of histone deacetylase complexes and HP1, are crucial to properly regulate the expression of KAP1-target genes in response to different extracellular stimuli [7,8,9].

In previous reports, we described the functional properties of ZNF224, a member of the KRAB-ZFP family. We demonstrated that ZNF224-mediated repression requires the recruitment of KAP1 and of the histone deacetylase HDAC1, in order to inhibit the human aldolase A gene transcription [10]. Moreover, we identified PRMT5, a type II protein arginine methyltransferase as a novel component of ZNF224 transcriptional repression complex and demonstrated that histone arginine methylation by PRMT5 is necessary for ZNF224-mediated gene repression [11].

These findings prompted us to explore the possibility that PRMT5 interacts with KAP1 and the role of PRMT5 on KAP1 methylation.

## 2. Results and Discussion

**Figure 1:**
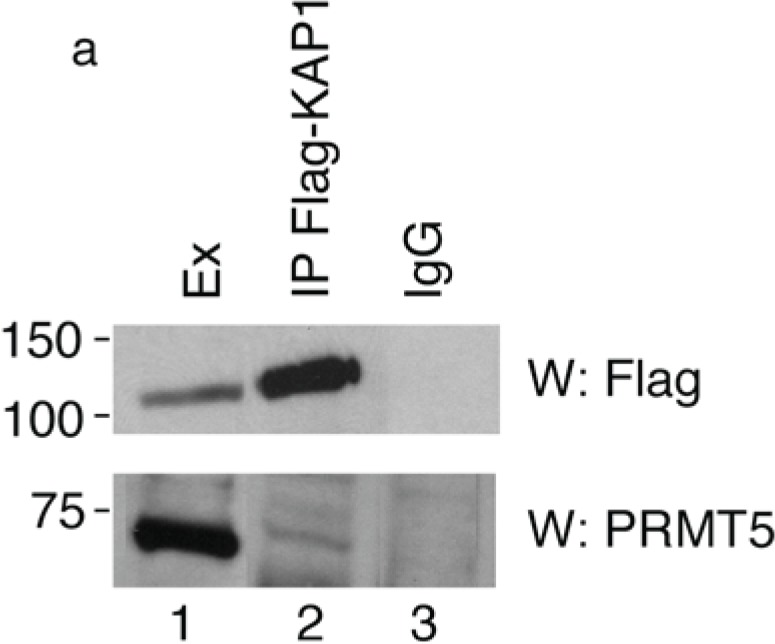

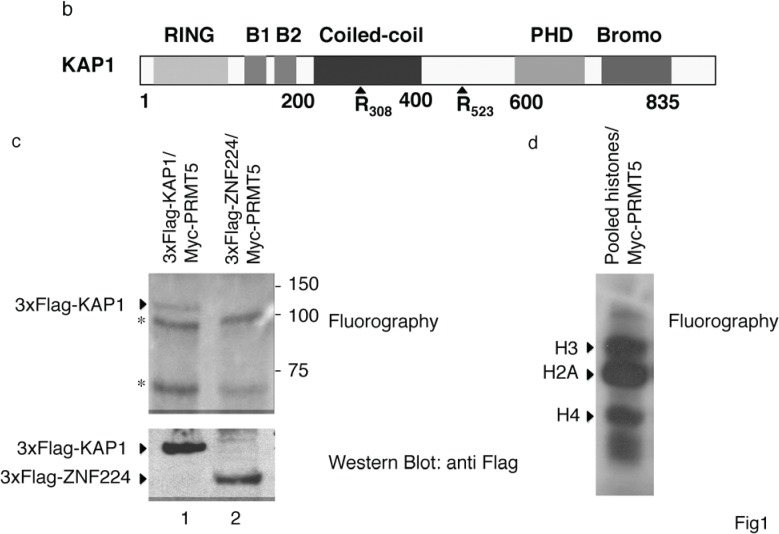
KAP1 interacts with PRMT5 and is a substrate for PRMT5-mediated methylation. (**a**) KAP1 interacts with PRMT5 protein. Flag-tagged KAP1 was immunoprecipitated from HEK293 cells with anti-Flag antibody or control immunoglobulin (IgG), as indicated. The specific interaction of KAP1 with the endogenous PRMT5 protein was detected by Western blot analysis with anti-PRMT5 antibody. Relative amount of immunoprecipitated Flag-KAP1 was visualized by Western blot analysis with anti-Flag antibody; (**b**) A schematic representation of the domains in the KAP1 protein. The arginine residues at positions 308 and 523 are potential methylation sites for PRMT5; (**c**) KAP1 is an *in vitro* substrate of PRMT5. The recombinant proteins Flag-KAP1 and Flag-ZNF224 were purified from hypomethylayed HEK293 cells and subjected to an *in vitro* methylation assay using recombinant Myc-PRMT5 and [3H]-AdoMet. The 3H-labeled proteins were resolved by SDS-PAGE on an 8% gel and visualized by fluorography. The asterisks indicate nonspecific bands. An aliquot of the substrates used in the *in vitro* methylation reaction were immunoblotted with anti-Flag antibody; (**d**) Pooled histones, used as positive control for the *in vitro* methylation assay, were visualized by fluorography. Results shown in Figure 1 are representative of two independent experiments.

To verify the interaction between PRMT5 and KAP1, we transfected HEK293 cells with a 3xFlag-KAP1 expression vector and performed a co-immunoprecipitation assay with Flag antibody followed by Western blotting with anti-PRMT5 antibody. We found that the endogenous PRMT5 protein specifically co-precipitated with the exogenously expressed KAP1 (Figure 1a, lower panel), thus indicating that KAP1 interacts with PRMT5.

Based on literature data reporting that PRMTs are associated with their substrates [12] and *in silico* sequence analysis of KAP1 protein revealing the presence of potential sites for PRMT5 methylation (motifs RG) [13] on the arginine residues at positions 308 and 523 (Figure 1b), we hypothesized that PRMT5 can methylate KAP1. To verify this hypothesis, we performed an *in vitro* methylation assay by using the recombinant protein Myc-PRMT5, immunoprecipitated from HEK293 cells, and the purified hypomethylated recombinant proteins Flag-KAP1 or, as negative control, Flag-ZNF224, which is not methylated by PRMT5 [11]. To generate hypomethylated protein extracts that are good *in vitro* substrates for methyltransferases, Flag-KAP1 and Flag-ZNF224 were purified from HEK293 cells cultured in the presence of adenosine periodate oxidized (AdOx) [14], an adenosylhomocysteine hydrolase inhibitor that causes the accumulation of intracellular adenosylhomocysteine (AdoHcy) levels. This increase in AdoHcy levels results in feedback inhibition of most methylation reactions. Indeed, a number of specific PRMT inhibitors recently identified have proven to be of limited use due to their inability to enter cells or their cytotoxic effects [15].

The hypomethylated recombinant proteins Flag-KAP1 and Flag-ZNF224 so obtained were incubated in presence of S-adenosyl-L-methyl-^3^H-methionine (^3^H-AdoMet). The incorporation of radiolabeled methyl group on Flag-KAP1 and Flag-ZNF224 was evaluated by fluorography (Figure 1c). As shown, a radioactive signal corresponding to the molecular weight of KAP1 was detected in the presence of Flag-KAP1 and PRMT5 (lane 1), whereas no specific bands were observed in the presence of Flag-ZNF224 and PRMT5 (lane 2), thus indicating that PRMT5 could specifically methylate KAP1 *in vitro*. Arginine methylation of pooled histones was used as a positive control of the *in vitro* methylation assay (Figure 1d).

**Figure 2:**
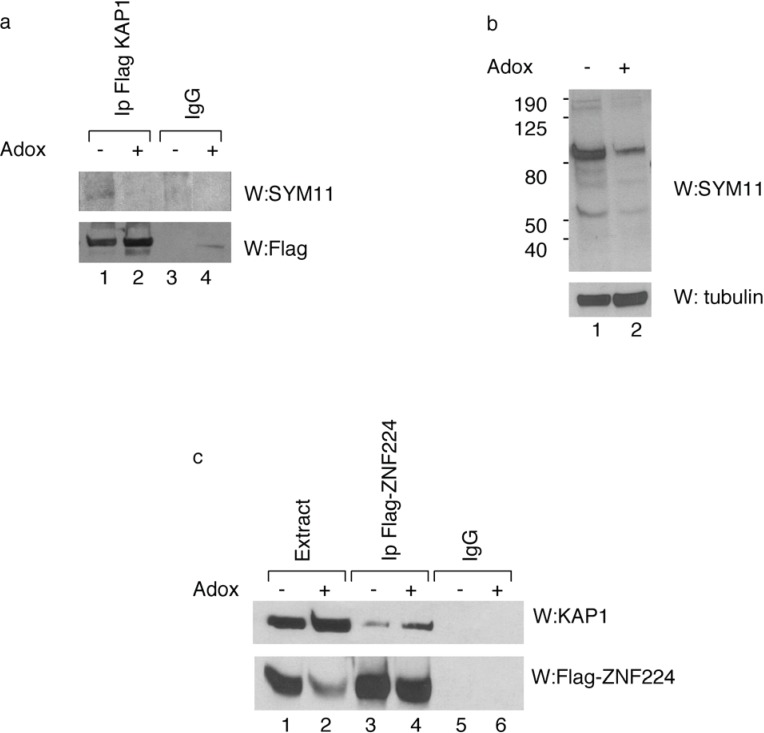
*In vivo* methylation of KAP1 affects its interaction with ZNF224. (**a**) Immunoprecipitation of methylated KAP1 protein. KAP1 exogenously expressed in HEK293 in the absence or presence of AdOx treatment was immunoprecipitated with anti-Flag antibody or control immunoglobulin (IgG), as indicated. After immunoprecipitation, Western blot analysis was performed with SYM11 antibody. A relative amount of immunoprecipitated Flag-KAP1 was visualized by Western blot analysis with anti-Flag antibody; (**b**) To verify the inhibition of protein methylation after 24h of AdOx treatment, whole cell extracts of HEK293 cells treated or untreated with AdOx were immunoblotted with anti-tubulin or SYM11 antibodies; (**c**) Interaction between KAP1 and ZNF224 is enhanced by AdOx treatment. Cell lysates from HEK293 cells transfected with Flag-ZNF224 in the absence or presence of AdOx treatment were immunoprecipitated with anti-Flag or anti-IgG antibodies; Western blot was performed using antibodies against KAP1 protein. A relative amount of immunoprecipitated Flag-ZNF224 was visualized by Western blot analysis with anti-Flag antibody. One representative experiment out of two performed is shown.

To determine the arginine methylation status of KAP1 *in vivo*, Flag-KAP1 protein was immunoprecipitated from HEK293 cells treated or untreated with AdOx. Western blot analysis with SYM11 antibody, which specifically recognizes the symmetric dimethylation of arginine, showed that the immunoprecipitated KAP1 was recognized by SYM11 antibody, thus confirming *in vivo* the KAP1 methylation status (Figure 2a, lane 1). As expected, KAP1 protein immunoprecipitated from hypomethylated cell extract had a low level of arginine residues methylation (Figure 2a, lane 2).

A growing body of evidence indicates that post-translational modifications may regulate KAP1 activity, leading to different biological effects and thus expanding the functional roles of KAP1. Moreover, protein arginine methylation of coactivators, corepressors and transcription factors plays important roles in transcriptional regulation, being able to affect protein-protein interaction, protein-DNA or protein-RNA interactions. [16].

In particular, methylation of arginine residues by PRMT5 can act either as negative or positive regulator of protein-protein interactions [17,18].

We observed that the RBCC domain of KAP1, which is necessary for interaction with the KRAB domain of KRAB-zinc finger proteins, contains a potential site (R308) for PRMT5 methylation. So, we speculated that the arginine methylation of KAP1 by PRMT5 could play a role in regulating KAP1 function.

In order to obtain further information about the effect of KAP1 methylation on KAP1/KRAB zinc finger protein interaction, the HEK293 cells were transfected with the expression vector for Flag-ZNF224 in presence or absence of AdOx treatment and analyzed by IP-Western blot. We observed that KAP1 co-precipitated with Flag-ZNF224 in a methylation-dependent fashion (Figure 2c). In fact, the KAP1/ZNF224 interaction was enhanced by AdOx treatment (Figure 2c, lane 4 *vs.* lane 3). This result indicates that arginine KAP1 methylation destabilizes of the association between KAP1 and ZNF224, thus suggesting that the interaction between the corepressor KAP1 and ZNF224 could be affected by PRMT5 catalytic activity.

We previously demonstrated the cell-cycle dependent recruitment of PRMT5 on the ZNF224 transcriptional repression complex [11]. This finding leads us to speculate that in certain genomic contexts KAP1 methylation, by preventing the interaction with ZNF224, could contribute to the switch of ZNF224 from a repression to a transcriptional activation complex. Indeed, a growing body of evidences indicates that, although traditionally described as transcriptional repressors, KRAB-ZFPs may actually play either positive or negative roles in transcription [19].

Moreover, arginine methylation could cross-talk with other KAP1 modifications such as phosphorylation and SUMOylation. Identification of methylation sites on KAP1 may provide a hint at the synergistic or antagonistic relationships among these modifications and may contribute to clarify the combinatorial effects of different post-translational modifications on a variety of biological processes in which KAP1 is involved.

## 3. Experimental Section

### 3.1. Cell Culture and Transient Transfection

HEK293 cells were maintained in Dulbecco’s modified Eagle’s medium (GIBGO-BRL) supplemented with 10% foetal bovine serum (FBS), 100 mg/mL penicillin, and 100 U/mL streptomycin at 37 °C in 5% CO_2_. For transfection, HEK293 cells were seeded at 1 × 10^6^ cells/ 10-cm plate and transiently transfected with 10 mg of 3xFlag-ZNF224, 3xFlag-KAP1 and Myc-PRMT5 expression vectors [10,11] by using Metafectene reagent (Biontex), as recommended by the manufacturer.

### 3.2. Co-Immunoprecipitation and Western Blotting Assays

To purify the recombinant proteins 3xFlag-ZNF224, 3xFlag-KAP1 and Myc-PRMT5, to be used for *in vitro* methylation assay, anti-Flag M2 agarose beads and anti-c-Myc agarose conjugate (10 mL beads/mg proteins, Sigma) were added to 10 mg of cell lysates, obtained with 50 mM Tris-HCl pH 8.0, 150 mM NaCl, 0.1% NP40, 10% glycerol, 0.5 mM phenylmethylsulfonyl fluoride (PMSF), 1 mg/mL aprotinin and 1 mg/mL pepstatin A; after incubation for 3 h at 4 °C, the immunoprecipitates were rinsed with Wash Buffer (50 mM Tris-HCl pH 8.0, 300 mM NaCl, 1 mM EDTA pH 8.0, 10% glycerol, 0.1% NP-40, 0.5 mM PMSF, 1 mg/mL aprotinin and 1 mg/mL pepstatin A), Cell Lysis buffer, and Methylation Buffer (50 mM TrisHCl, 1 mM EDTA, 1 mM EGTA, protease inhibitors). The recombinant proteins 3xFlag-ZNF224 and 3xFlag-KAP1 were competitively eluted from beads by affinity chromatography using Pure Yield Binding Columns (Promega) and the peptide Flag 100 µg/mL (Sigma) in 50 µL of Methylation Buffer.

Also, to immunoprecipitate 3xFlag-ZNF224 and 3xFlag-KAP1 recombinant proteins to be used for *in vivo* analysis of arginine methylation and coimmunoprecipitation experiments, 5 µg of anti-Flag antibody (anti Flag-M2, Sigma) or 5 µg of IgG were added to 1mg of protein extract, precleared with 25 mL of A/G Plus Agarose (Santa Cruz) for 2 h. Immunoprecipitations were carried out overnight at 4 °C. The immune complexes were collected with Protein A/G PLUS Agarose, washed with the lysis buffer and loaded on a 8% SDS-PAGE for western blot analysis with anti-Flag (anti Flag-M2, Sigma, 1:5000 dilution), anti-KAP1 (Novus Biologicals, 1:1000 dilution), anti-SYM11 and anti-PRMT5 antibodies (Upstate, 1 mg/mL).

#### AdOx Treatment of Cultured Cells

For adenosine periodate oxidized (AdOx) treatment, HEK293 cells were incubated with 2 mM AdOx 24 h after transfection with 3xFlag-KAP1 or 3xFlag-ZNF224 plasmids and cultured for another 24 h. AdOx treatment for 24 h generates hypomethylated protein extracts that are good substrates for *in vitro* methylation labeling or *in vivo* analysis of arginine methylation.

### 3.3. In Vitro Methyltransferase Assay

The methyltransferase assay was performed according to the protocol of Amente *et al.* [20], using 3xFlag-ZNF224, 3xFlag-KAP1 and Myc-PRMT5 recombinant proteins immunopurified as described above.

### 3.4. In Vivo Analysis of Arginine Methylation

The recombinant protein 3xFlag-KAP1, expressed in HEK293 cells treated or untreated with AdOx, was immunoprecipitated with anti-Flag antibody as described above and loaded on an 8% SDS-PAGE for Western blot analysis with SYM11 antibody.

## 4. Conclusions

In this study, we show that arginine methylation is a novel KAP1 post-translational modification and suggest that this modification could affect the interaction between the corepressor KAP1 and the KRAB zinc finger protein ZNF224. Our preliminary findings contribute to gainnovel insight into the molecular mechanisms underlying KRAB-ZFP-mediated repression. However, further investigations will be necessary to identify the methylation sites in KAP1 and to better clarify how this post-translational modification regulates KAP1 function.
